# National High Blood Pressure Education Month — May 2014

**Published:** 2014-05-02

May is National High Blood Pressure Education Month. High blood pressure, also known as hypertension, is the leading risk factor for stroke and a major cause of morbidity and mortality (1). In the United States, nearly one in three adults has hypertension, but only about half (47%) of those have it under control (1). Hypertension is considered the “silent killer” because it can damage the heart, brain, and kidneys without any symptoms (1). Each day in the United States, nearly 1,000 deaths are associated with hypertension (2). National High Blood Pressure Education Month aims to save lives by increasing awareness and educating the public about cardiovascular risks and how to prevent them.

To control hypertension, patients can take medications as directed, measure their blood pressure, and eat a lower-sodium diet and more fruits and vegetables (1). Health-care providers can use electronic health records, blood pressure monitoring, and a team-based care approach to help improve their patients’ hypertension control (3).

CDC’s Division for Heart Disease and Stroke Prevention focuses on promoting cardiovascular health and improving quality of care for all and eliminating disparities associated with heart disease and stroke. Additional information is available at http://www.cdc.gov/bloodpressure and http://www.cdc.gov/stroke.
